# The Potential Mechanisms Involved in the Disruption of Spermatogenesis in Mice by Nanoplastics and Microplastics

**DOI:** 10.3390/biomedicines12081714

**Published:** 2024-08-01

**Authors:** Yixian Wen, Jing Cai, Huilian Zhang, Yi Li, Manyao Yu, Jinyi Liu, Fei Han

**Affiliations:** 1 School of Public Health, Chongqing Medical University, Chongqing 400016, China; 191079@cqmu.edu.cn (Y.W.); caij@stu.cqmu.edu.cn (J.C.); zhanghl629@163.com (H.Z.); 2023121669@stu.cqmu.edu.cn (Y.L.); 13814484117@163.com (M.Y.); 2 Joint International Research Laboratory of Reproduction and Development of the Ministry of Education, Chongqing 400016, China; 3 Institute of Toxicology, College of Preventive Medicine, Army Medical University, Chongqing 400038, China; jinyiliutmmu@163.com

**Keywords:** nanoplastics, microplastics, reproductive toxicity, spermatogenesis, mechanism

## Abstract

Background: Plastic-based products are ubiquitous due to their tremendous utility in our daily lives. Nanoplastic (NP) and microplastic (MP) pollution has become a severe threat to the planet and is a growing concern. It has been widely reported that polystyrene (PS) MPs are severely toxic to the male reproduction system, with effects including decreased sperm parameters, impaired spermatogenesis, and damaged testicular structures. However, the molecular mechanisms for impaired spermatogenesis remain poorly understood. Methods: C57BL/6 male mice were treated with PS-NPs (80 nm) and PS-MPs (5 μm) by oral gavage every day for 60 days. A series of morphological analyses were completed to explore the influence of PS-NP and PS-MP exposure on the testes. Compared to other cell types in the seminiferous tubule, PS-NP and PS-MP exposure can lead to decreased spermatocytes. Then, more refined molecular typing was further performed based on gene expression profiles to better understand the common and specific molecular characteristics after exposure to PS-NPs and PS-MPs. Results: There were 1794 common DEGs across the PS-NP groups at three different doses and 1433 common DEGs across the PS-MP groups at three different doses. GO and KEGG analyses of the common DEGs in the PS-NP and PS-MP groups were performed to enrich the common and specific functional progress and signaling pathways, including 349 co-enriched GO entries and 13 co-enriched pathways. Moreover, 348 GO entries and 33 pathways were specifically enriched in the PS-NP group, while 526 GO entries and 15 pathways were specifically enriched in the PS-MPs group. Conclusions: PS-NPs were predominantly involved in regulating retinoic acid metabolism, whereas PS-MPs primarily influenced pyruvate metabolism and thyroid hormone metabolism. Our results highlight the different molecular mechanisms of PS-NPs and PS-MPs in the impairment of spermatogenesis in male mammals for the first time, providing valuable insights into the precise mechanisms of PS-NPs and PS-MPs in male reproduction.

## 1. Introduction

During the last twenty years, plastic environmental pollution has grown, affecting terrestrial and aquatic ecosystems and the atmosphere and threatening living organisms [1,2]. Abandoned plastic products are broken down into particles through a series of physical, chemical, and biodegradation processes [3]. Plastics in the range of 100 nm to 5 mm are defined as microplastics (MPs), and those in the range of 1–100 nm are defined as nanoplastics (NPs), which have been detected mainly in oceans, sediments, sewage, and rivers and on urban beaches [4]. These low-diameter plastics can pass from the lower nutrient levels to the higher nutrient levels through the food chain, ultimately threatening human health [5,6]. Recently, studies have shown the presence of MPs and NPs in human blood, lungs, feces, semen, and placentas [7,8,9,10,11]. Previous studies show that exposure to 5–150 μm MPs in the intestines of mice induces inflammation, alters the microbiome, influences liver lipid metabolism, and causes gastrointestinal toxicity and hepatotoxicity [12,13]. Compared to MPs, 293 nm NPs can cross the blood–brain barrier. Moreover, NPs can combine with α-synuclein fibrils to exacerbate the spread of α-synuclein pathology across interconnected vulnerable brain regions, stimulating Parkinson’s disease-associated features in the brain [14,15]. Mice exposed to 20 nm, 0.5 μm, 4 μm, and 10 μm MPs and NPs by oral gavage have shown the accumulation of MPs and NPs in the testes, corresponding with histological changes and decreased serum hormone levels, which are essential for germ cell development [16,17,18]. Furthermore, pregnant mice exposed to 10 μm MPs have shown increased resorption rates of embryos and decreased placental diameters, which compromises embryo development [19]. These studies have identified that MPs and NPs can cause adverse effects on health, including reproductive dysfunction.

The reproductive system is one of the most sensitive systems of an organism that can be affected by environmental pollution. Male infertility, a serious reproductive health problem for humans, is gaining attention as it poses a threat to human reproductive safety with the ongoing deterioration of the environment. The normal concentration and sperm count of young men have decreased over the past years [20]. Plastic particles are considered one of the key factors leading to the declining reproductive capacity of males. The testes are vital reproductive and endocrine organs made up of seminiferous tubules and interstitial spaces. The primary function of testes is to produce sperm through a process called spermatogenesis [21]. This process is complex and involves the development of male germ cells from spermatogonia to spermatocytes and finally to spermatids within the seminiferous tubules. Any interference caused by environmental pollution can have adverse effects on sperm parameters [22]. Studies have indicated that MPs are harmful to the male reproductive system of mammals. Twenty-nanometer polystyrene nanoplastics (PS-NPs) can harm testosterone synthesis and male reproductive function [18]. Exposure to 0.5 μm, 4 μm, and 10 μm PS-MPs can lead to testicular inflammation and disruption of the blood–testes barrier [16]. In our previous study, it was noted that exposure to 80 nm and 5 μm PS-MPs for 60 days resulted in altered testicular histology, decreased serum testosterone levels, and declined sperm quality, ultimately leading to male reproductive dysfunction in mice [23]. However, the precise effects and underlying mechanisms of PS-NP and PS-MP exposure on the process of spermatogenesis are still largely unexplored.

MP/NP toxicity can be influenced by various factors, including the type of plastic, particle size, environmental conditions, species, experimental model, exposure time, route, and concentration [24,25,26,27]. PS-NPs (50 nm) enter testes cells via endocytosis and affect microstructures [28]. Recent research has shown that compared to NP exposure, MPs lead to a more significant increase in reproductive toxicity, dependent on particle size [29]. In our previous study, we discovered that exposure to PS-MPs resulted in the death of spermatogenic cells through apoptosis [23]. However, the differences in the molecular characteristics and pathways between PS-NP and PS-MP exposure in germ cells are still not understood. 

In this study, we established both in vivo and in vitro models to study the effects of various doses of PS-MPs and PS-NPs on spermatogenesis. The experimental design was based on previous research [23]. We aimed to evaluate the impact of different sizes of PS plastics on male reproductive function by assessing cell type changes and identifying the different DEGs between the PS-MP and PS-NP exposure groups, as well as by determining the key common and specific differential molecules and signaling pathways. To gain a better understanding of the potential mechanisms, we further confirmed the key DEG changes after PS-MP and PS-NP exposure. 

## 2. Materials and Methods

### 2.1. Microplastic Particle 

The polystyrene microplastic particles (nominal diameter, 5 μm, and 80 nm; purity > 98%) in a vial concentration of 5% *w*/*v* and a 10 mL capacity were purchased from Tianjin Baseline Technology Research Center (Tianjing, China). The polystyrene microplastic particles were dispersed in sterile water before the experiments. 

### 2.2. Animals and Treatment 

Six-week-old male C57/BL6 mice (18–20 g) were purchased from Chongqing Medical University Lab Animal Centre (Chongqing, China, Certificate: SICXK (YU) 2007-0001) and were used following the guidelines of Institutional Animal Care and Use Committee of Chongqing Medical University (Chongqing, China). This research received approval from the Ethics Committee (approval number: IACUC-CQMU-2023-0458) of Chongqing Medical University. Mice were placed in the animal room under specific pathogen-free conditions and allowed to acclimate for one week. They were maintained under standard conditions of 22 ± 2 °C, 50–60% humidity, with a 12 h light-dark cycle. After being domesticated one week, the mice were randomly divided into three groups, each consisting of twelve mice. Two groups were given either 5 μm or 80 nm microplastic particles at a dose of 40 mg/kg/d. The third group was given ultra-pure water as a control. The mice received oral gavage every day for 60 days, as reported previously [23]. The testes tissues were collected from each group and fixed in testicular-tissue fixation fluid (Wuhan Servicebio Biotechnology Co., Ltd., Wuhan, China, Cat, G1121-15ML) for 4–6 h at 26 °C, and then they were subsequently embedded in paraffin. Afterward, sections were cut into a thickness of 5 μm for hematoxylin and eosin (H&E) staining and finally visualized under an Olympus microscope (BX40, Olympus, Tokyo, Japan).

### 2.3. Cell Culture and Treatment

The GC-2 cell line was obtained from the American Type Culture Collection (ATCC, Manassas, VA, USA) and cultured in DMEM medium with 10% FBS and maintained in a 5% CO_2_ incubator at 37 °C. The culture medium was refreshed every 48 h to ensure optimal growth. Logarithmic growth phase cells were used for the experiment. The polystyrene nanoplastic and microplastic particles were divided into three different dose groups, with concentrations of 10 μg/mL, 20 μg/mL, and 40 μg/mL. The control group used ultra-pure water for comparison. After 72 h of exposure, the cells from each group were collected for transcriptome sequencing and real-time qPCR (RT-qPCR) analysis.

### 2.4. Transcriptome Sequencing

The high-throughput transcriptome sequencing of GC-2 cells in different groups was conducted as previously described [22]. Briefly, the RNA was isolated from GC-2 cells using the TRIZOL method. The Bioanalyzer 2100 and Agilent RNA 6000 Nano LabChip kit (Agilent, CA, USA) were used to check the RNA integrity, quality quickly, and RNA concentration accurately. Library construction was performed using the PrimeScript^®^ RT regent kit with the gDNA Eraser kit. The HISAT2 software was used as the reference genome mapping tool, and the Deseq2 software was used to analyze the differential expression between the different groups. 

### 2.5. DEG Screening Analysis 

GC-2 cells of varying doses and sizes were compared to the control group. Genes that did not have matching names were eliminated, and data that were less than zero were rectified. The R package called limma was utilized to find DEGs in version 4.0. Before conducting the difference analyses, the RNAseq-counts data underwent normalization. |Fold change| > 2 was the criterion for screening. To obtain their DEGs, genes that did not have corresponding ENTREZIDs and gene symbols were removed after Gene ID was converted to ENTREZIDs and gene symbols. A Venn diagram was utilized to ascertain the shared and unique DEGs across the various dosage groups, using the DEG profiles as a basis. The website of jvenn (https://jvenn.toulouse.inrae.fr/app/index.html (accessed on 21 July 2024)) was used to create the Venn diagram [30]. 

### 2.6. Functional Analysis of the DEGs

The GO plot and cluster Profiler programs in R 4.0 were used to conduct the Gene Ontology (GO) pathway enrichment of DEGs, and the Kyoto Encyclopedia of Genes and Genomes (KEGG) conversion was shown. When *p* < 0.05, it was discovered that the KEGG and GO pathways were significantly enhanced. The top ten GO terms and KEGG enrichment pathways were shown using the ggplot2 tools in R 4.0.

### 2.7. Real-Time qPCR

The total RNAs of GC-2 cells were extracted using RNAiso plus reagent following the manufacturer’s instructions after a 72 h treatment. We used a 10 μL reaction system with an RT-qPCR kit obtained from TaKaRa Biotechnology in Dalian, China, to synthesize cDNA after treating the whole pool of RNAs with DNase I. Table 1 lists the primers for genes. After using SYBR Green for transcript quantification, the Bio-Rad CFX Manager 3.1 Detection System (USA) was brought into play for PCR. The relative gene expression in various tissue samples was determined using the 2^−ΔΔCt^ technique, with β-actin serving as the internal reference. Each sample was run in triplicate for the experiments. 

### 2.8. Statistical Analysis

The GraphPad Prism version 9.0 software (Graph-Pad Software Inc., La Jolla, CA, USA) was used for data analysis, with Student’s *t*-test used for data comparison for two groups and two-way analysis of variance (ANOVA; Kruskal–Wallis test) used for comparisons of multiple groups. A *p*-value of less than 0.05 was considered significant. All data are reported as mean ± standard deviation (SD).

## 3. Results

### 3.1. PS-MP and PS-NP Exposure Reduced Spermatocytes In Vivo

**Figure 1:**
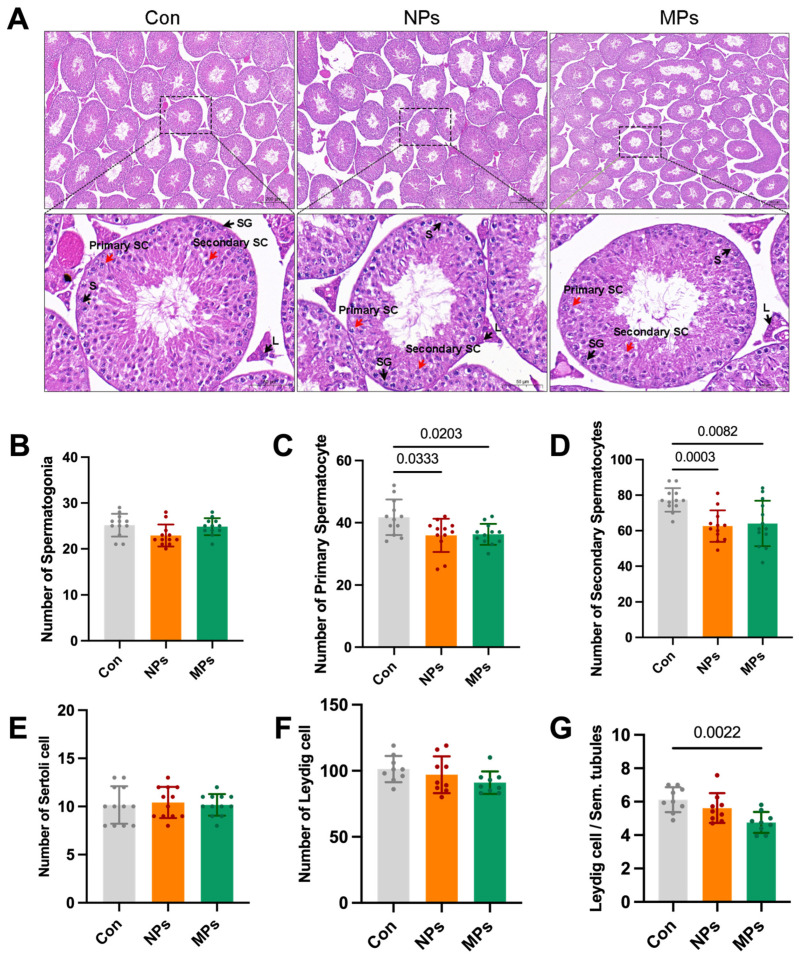
Effects of PS-NPs and PS-MPs on testes and each type of germ cell. (**A**) Histological sections and H&E staining (scale bar = 200 μm and 50 μm) of testicular tissue collected after exposure to PS-NPs and PS-MPs. (**B**) The average number of spermatogonia in each seminiferous tubule. (**C**) The average number of primary spermatocytes in each seminiferous tubule. (**D**) The average number of secondary spermatocytes in each seminiferous tubule. (**E**) The average number of Sertoli cells in each seminiferous tubule. (**F**) The average number of Leydig cells in each seminiferous tubule. (**G**) The ratio of Leydig cells to seminiferous tubules in 3 random fields of view in each testis. SG, spermatogonia; SC, spermatocytes; S, Sertoli cell; L, Leydig cell.

The testicles are the most important site for spermatogenesis and the target organ for reproductive toxicity. To clarify the effect of PS-MP and PS-NP exposure on the process of spermatogenesis, we first determined the morphological changes in the testes. Histopathological changes in the testes showed a loose structure and decreased spermatocytes in the PS-MP and PS-NP groups (Figure 1A). A quantitative analysis of the cellular composition was conducted in the seminiferous tubules among the control, PS-NP, and PS-MP groups. The results showed that the number of primary spermatocytes decreased by 13.9% in the PS-NP group and by 13.1% in the PS-MPs group (*p* < 0.05) (Figure 1C). The number of secondary spermatocytes decreased by 19.1% in the PS-NP group and by 17.1% in the PS-MP group (*p* < 0.05) (Figure 1D). However, the number of other cell types did not significantly change. These data suggest that spermatocytes are more susceptible to interference from PS-NPs and PS-MPs.

### 3.2. PS-NP and PS-MP Exposure Altered the Transcriptome of Spermatocytes

**Figure 2:**
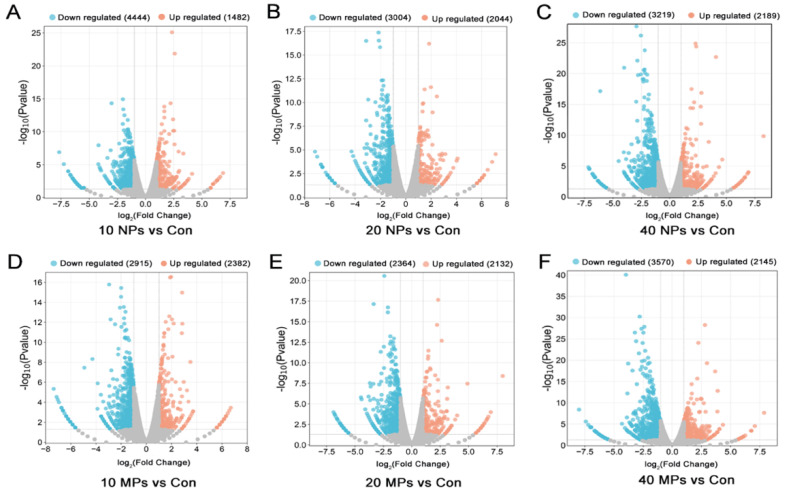
Volcano map of DEGs between the control group and different treatment groups. (**A**) The DEGs in the 10 μg/mL PS−NP group. (**B**) The DEGs in the 20 μg/mL PS−NP group. (**C**) The DEGs in the 40 μg/mL PS−NP group. (**D**) The DEGs in the 10 μg/mL PS−MP group; (**E**) The DEGs in the 20 μg/mL PS−MP group. (**F**) The DEGs in the 40 μg/mL PS−MP group. Orange dots denote upregulated genes and blue dots denote downregulated genes screened based on |Fold change| > 2; grey dots denote genes with no significant difference.

To unveil the mechanism of PS-MP- and PS-NP-induced damage of Spermatocytes in detail, we analyzed the transcriptome of GC-2 cells in different doses and sizes using high-throughput transcriptome sequencing. A comparative gene expression analysis was performed between the control and treatment groups in different microplastic particle doses and sizes, and the significantly differentially expressed genes (DEGs) were screened. Compared to the control group, 4444 low-expression genes and 1482 high-expression genes were observed in the 10 μg/mL PS-NP group; 3004 low-expression genes and 2044 high-expression genes were found in the 20 μg/mL PS-NP group; 3219 low-expression genes and 2189 high-expression genes were observed in the 40 μg/ mL PS-NP group; 2915 low-expression genes and 2382 high-expression genes were screened out in the 10 μg/mL PS-MP group; 2364 low-expression genes and 2132 high-expression genes were found in the 20 μg/mL PS-MP group; and 3570 low-expression genes and 2145 high-expression genes were observed in the 40 μg/mL PS-MP group (Figure 2A–F). The comparative analysis data suggest that the molecular characteristics of the treatment groups are significantly different from those in the control group. 

**Figure 3:**
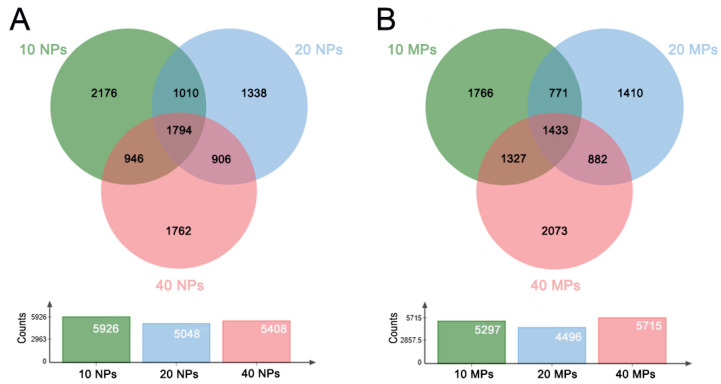
Venn diagram of common and specific DEGs in different groups. (**A**) Common and specific DEGs in the PS−NPs groups; (**B**) Common and specific DEGs in the PS−MPs groups.

To analyze the common and specific DEGs in the PS-NP and PS-MP groups, we performed an intersection on the Venn diagram. There were 1794 common DEGs across the PS-NP groups at three different doses and 1433 common DEGs in the PS-MP groups across three different doses. There were 2176 specific DEGs for the 10 μg/mL PS-NP group, 1338 specific DEGs for the 20 μg/mL PS-NP group, 1762 specific DEGs for the 40 μg/mL PS-NP group, 1766 specific DEGs for the 10 μg/mL PS-MP group, 1410 specific DEGs for the 20 μg/mL PS-MP group, and 2073 specific DEGs for the 40 μg/mL PS-MP group (Table 2; Figure 3A,B). The details of the common DEGs across the different groups and the specific DEGs for each group are shown in Table S1. These results suggest that polystyrene microplastics of different doses and sizes induce gene expression differently.

### 3.3. The Common and Specific GO Entries and KEGG Pathways in the PS-NP and PS-MP Groups

**Figure 4:**
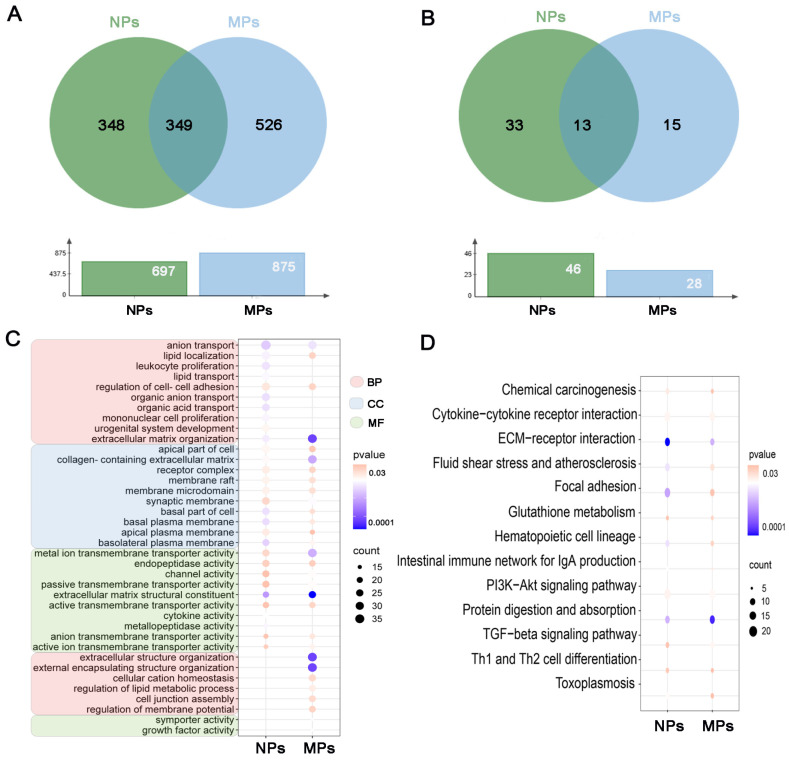
The common and specific GO entries and KEGG pathways between the PS−NP group and PS−MP group. (**A**) Venn diagram of common and specific GO terms in the PS−NP group and PS−MP group. (**B**) Venn diagram of common and specific KEGG pathways in the PS−NP group and PS−MP group. (**C**) The top 10 entries that are common between the PS−NP group and PS−MP group. (**D**) The common KEGG pathways between the PS−NP group and PS−MP group.

Common DEGs identified at different doses were used to predict the common molecular characteristics and pathways of the PS-NP and PS-MP groups. The functions of the DEGs in the treatment groups were predicted using GO enrichment and KEGG pathway analyses. The results of the GO enrichment analysis are classified into three categories: biological process (BP), cellular component (CC), and molecular function (MF). There were 349 co-enriched GO entries for the DEGs of the PS-NP and PS-MP groups (Figure 4A, Table S2). The top five biological processes in the PS-NP group were “anion transport”, “lipid localization”, “leukocyte proliferation”, “lipid transport”, and “regulation of cell–cell adhesion”. The top five biological processes in the PS-MP group were “extracellular matrix organization”, “extracellular structure organization”, “external encapsulating structure organization”, “anion transport”, and “cellular cation homeostasis”. The top five cellular components in the PS-NP group were the “apical part of the cell”, “collagen-containing extracellular matrix”, “receptor complex”, “membrane raft”, and “membrane microdomain”. The top five cellular components in the PS-MP group were “collagen-containing extracellular matrix”, “synaptic membrane”, “membrane raft”, “membrane microdomain”, and “receptor complex”. As shown in Figure 4C, the top five molecular functions in the PS-NP and PS-MP groups were “metal ion transmembrane transporter activity”, “endopeptidase activity”, “channel activity”, “passive transmembrane transporter activity”, and “extracellular matrix structural constituent”. Thirteen pathways were identified for co-enrichment of the DEGs in the PS-NP and PS-MP groups (Figure 4B). These pathways were related to “chemical carcinogenesis”, “cytokine–cytokine receptor interaction”, “ECM–receptor interaction”, “fluid shear stress and atherosclerosis”, “focal adhesion”, “glutathione metabolism”, “hematopoietic cell lineage”, “intestinal immune network for IgA production”, “PI3K-Akt signaling pathway”, “protein digestion and absorption”, “TGF-beta signaling pathway”, “Th1 and Th2 cell differentiation”, and “toxoplasmosis” (Figure 4D). These data suggest that different sizes of polystyrene microplastics have the same effects on GC-2 cells.

### 3.4. The Special GO Entries and KEGG Pathways in the PS-NP Group

**Figure 5:**
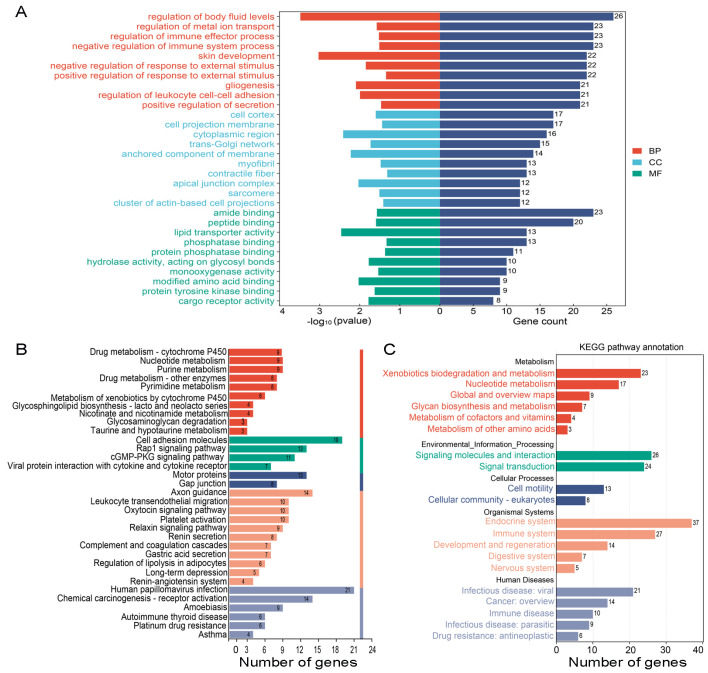
GO analysis and KEGG pathway analysis of the DEGs in the PS−NP group. (**A**) GO analysis of GC−2 cells in the PS−NP group. (**B**) KEGG pathway analysis of GC−2 cells in the PS−NP group. The red bar represents metabolism, the green bar represents environmental information processing, the blue bar represents cellular processes, the orange bar represents organismal systems, the grey bar represents human diseases. (**C**) Secondary classification of all KEGG pathways in the PS−NP group.

Next, we conducted a detailed analysis of the molecular characteristics and pathways, specifically in the PS-NP group. Comparative analysis of the PS-NPs group’s GO entries showed that a total of 348 GO entries were enriched for the DEGs, and these GO entries mainly concentrated in “retinol metabolism”, “retinoic acid metabolism”, “cell cycle”, and “the process of fertilization” were enriched in the PS-NP group. The top five biological processes were “regulation of body fluid levels”, “regulation of metal ion transport”, “regulation of immune effector process”, “negative regulation of immune system process”, and “skin development”. The top five cellular components were the “cell cortex”, “cell projection membrane”, “cytoplasmic region”, “trans-Golgi network”, and “anchored component of membrane”. The top five molecular functions were “amide binding”, “peptide binding”, “lipid transporter activity”, “phosphatase binding”, and “protein phosphatase binding” (Figure 5A). The detailed results of all enriched GO entries are shown in Table S3. A total of 33 KEGG pathways were enriched in the PS-NP group, mainly focusing on “purine metabolism”, “cell adhesion molecules”, and “Rap1 signaling pathway”. The top 10 KEGG pathways were “human papillomavirus infection”, “cell adhesion molecules”, “axon guidance”, “chemical carcinogenesis-receptor activation”, “motor proteins”, “Rap1 signaling pathway”, “cGMP-PKG signaling pathway”, “leukocyte transendothelial migration”, “oxytocin signaling pathway”, and “platelet activation” (Figure 5B). We then performed a secondary classification of all KEGG pathway annotations. These 33 KEGG pathways were mainly categorized into five terms: metabolism, environmental information processing, cellular processes, organismal systems, and human diseases. For the metabolism, the KEGG pathways were mainly enriched in “xenobiotic biodegradation and metabolism”, “nucleotide metabolism”, and “metabolism of cofactors and vitamins”. For the cellular process category, the KEGG pathways were mainly enriched in “cell motility” and “cellular community-eukaryotes”.

### 3.5. The Special GO Entries and KEGG Pathways in the PS-MP Group

**Figure 6:**
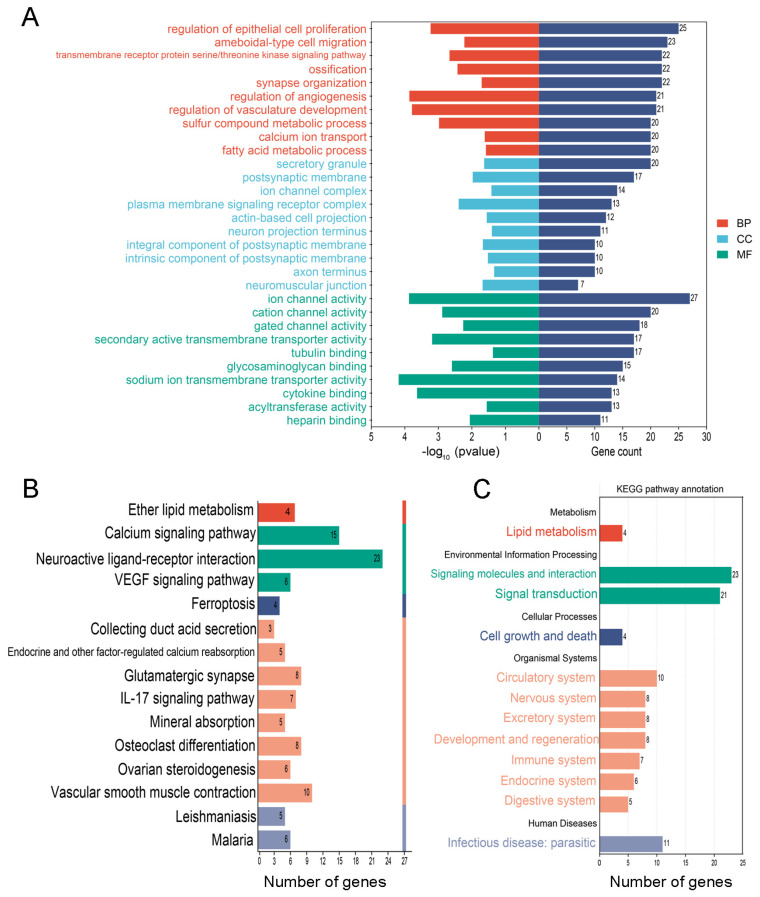
GO analysis and KEGG pathway analysis of the DEGs in the PS−MP group. (**A**) GO analysis of GC−2 cells in the PS−MP group. (**B**) KEGG pathway analysis of GC−2 cells in the PS−MP group. The red bar represents metabolism, the green bar represents environmental information processing, the blue bar represents cellular processes, the orange bar represents organismal systems, the grey bar represents human diseases. (**C**) Secondary classification of all KEGG pathways in the PS−MP group.

A total of 526 GO entries were enriched for the DEGs in the PS-MP group, and the specifically enriched GO entries were mainly concentrated in “angiogenesis”, “germ cell development”, “pyruvate metabolism”, “thyroid hormone metabolism”, and “iron ion homeostasis”. The top five biological processes were “regulation of epithelial cell proliferation”, “ameboidal-type cell migration”, “transmembrane receptor protein serine/threonine kinase signaling pathway”, “ossification”, and “synapse organization”. The top five cellular components were the “secretory granule”, “postsynaptic membrane”, “ion channel complex”, “plasma membrane signaling receptor complex”, and “actin-based cell projection”. The top five molecular functions were “ion channel activity”, “cation channel activity”, “gated channel activity”, “secondary active transmembrane transporter activity”, and “tubulin binding” (Figure 6A, Table S4). A total of 15 KEGG pathways were enriched for the DEGs in the PS-MP group, and the specifically enriched pathways mainly focused on “ferroptosis”, “VEGF signaling pathway”, and “cell differentiation”. The top 10 KEGG pathways were “neuroactive ligand–receptor interaction”, “calcium signaling pathway”, “vascular smooth muscle contraction”, “glutamatergic synapse”, “osteoclast differentiation”, “IL-17 signaling pathway”, “malaria”, “ovarian steroidogenesis”, “VEGF signaling pathway”, and “endocrine and other factor-regulated calcium reabsorption” (Figure 6B). The secondary classification of all KEGG pathway annotations in the PS-MP group showed that the pathway was mainly enriched in “lipid metabolism” and “cell growth and death” (Figure 6C). These results suggest that different sizes of polystyrene microplastics probably cause alterations in spermatocytes through different molecular mechanisms.

### 3.6. Screening and Validation of the Key Genes from the DEGs in the PS-NP and PS-MP Groups

**Figure 7:**
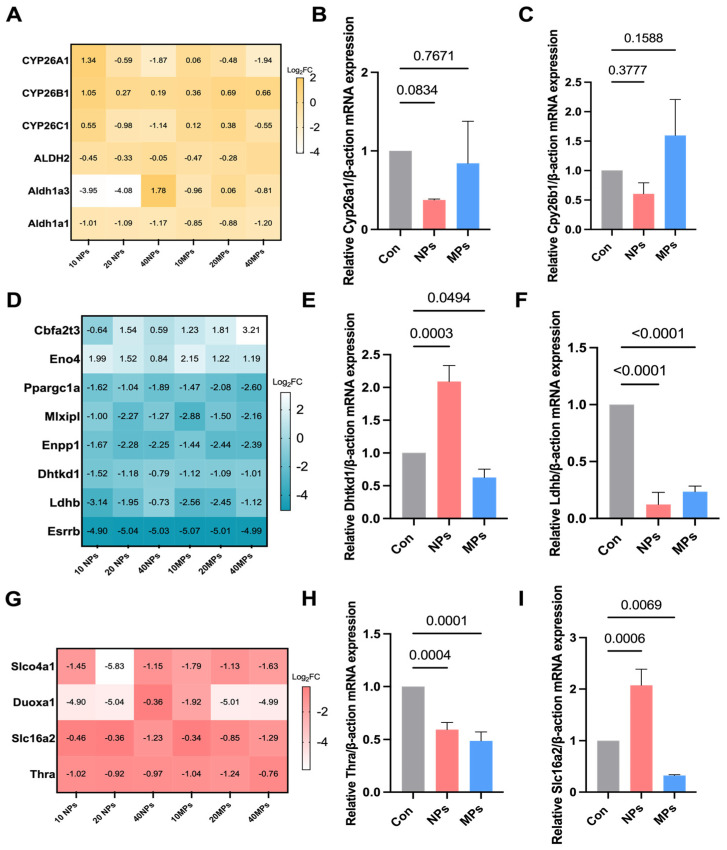
The validation of essential functional and mechanistic characteristics in the PS−NP group and PS−MP group. (**A**) The heat maps of different genes associated with the retinoic acid metabolism. (**B**) Relative expression of Cyp26a1 mRNA in GC-2 cells exposure to PS−NPs and PS−MPs. (**C**) Relative expression of Cyp26b1mRNA in GC−2 cell exposure to PS−NPs and PS−MPs. (**D**) The heat maps of different genes associated with the pyruvate metabolism. (**E**) Relative expression of Dhtkd1 mRNA in GC−2 cell exposure to PS−NPs and PS−MPs. (**F**) Relative expression of Ldhb mRNA in GC−2 cell exposure to PS−NPs and PS−MPs. (**G**) The heat maps of different genes involved in the thyroid hormone metabolism. (**H**) Relative expression of Slc16a2 mRNA in GC−2 cell exposure to PS−NPs and PS−MPs. (**I**) Relative expression of Thra mRNA in GC−2 cell exposure to PS−NPs and PS−MPs.

Based on the above enrichment analysis, we speculate that “retinoic acid metabolism”, “pyruvate metabolism”, and “thyroid hormone metabolism” may play key roles in the effects of the PS-NP and PS-MP groups. To fully characterize which genes are the core targets after PS-NP or PS-MP exposure, we identified genes related to retinoic acid, pyruvate, and thyroid hormone metabolism in the different GC-2 cell treatments. The results showed that the retinoic-acid-metabolism-related gene Cyp26b1 was upregulated while the other genes were downregulated (Figure 7A), as the retinoic-acid-degrading enzyme Cyp26a1 was downregulated after PS-NP exposure (Figure 7B,C). The results also showed that the pyruvate-metabolism-related genes Cbfa2t3 and Eno4 were upregulated while the other genes were downregulated (Figure 7D). As phosphonate analogs, the Dhtkd1-encoded 2-oxoadipate dehydrogenase (OADH) oxidizes 2-oxoadipate-a and pyruvate were established as specific inhibitors of cognate 2-oxo acid dehydrogenases [31,32], which were significantly downregulated after PS-MP exposure (Figure 7E). Lactate dehydrogenase B (LdhB), as an enzyme that catalyzes the conversion of lactate to pyruvate [33], was also significantly downregulated after PS-MP exposure (Figure 7F). Regarding the thyroid-hormone-metabolism-related genes, they were abnormally expressed at low levels in the PS-NP and PS-MP groups (Figure 7G). The thyroid hormone receptor alpha (Thra)-encoded thyroid hormone receptors (THRs), which predominantly mediate thyroid hormone action, were significantly downregulated after PS-NP and PS-MP exposure (Figure 7H). Slc16a2 is a specific thyroid hormone (T4 and T3) transporter [34] that was downregulated after exposure to PS-MPs but upregulated after exposure to PS-NPs (Figure 7I). These data suggest that PS-NP and PS-MP exposure induces various changes in molecular characteristics.

## 4. Discussion

Currently, the main plastic types are polyethylene, polyvinyl chloride, polystyrene, and polypropylene. Polystyrene (PS) is widely used in optical instruments, the chemical industry, daily necessities, and food packaging [35]. The whole population is extensively exposed to PS-MPs, which accumulate in mammalian tissues. Previous studies have shown that PS-MPs can cause harm to various organs in animal models, including the liver, gut, lungs, and reproductive system [36,37,38]. The male reproductive system is invariably considered highly susceptible to environmental factors. Recent studies have shown that microplastics can have negative effects on male fertility and sperm quality [18,23]. Nanoscale plastics are commonly believed to be more harmful than micrometer-scale plastics because they can cross the blood–testes barrier more easily [39]. There has been limited research on the impact of nanoplastics on the male reproductive system compared to microplastics. As reported in our previous study, 60 days is not only two consecutive spermatogenesis cycles but also the time of sub-chronic exposure to microplastics that can lead to male reproductive toxicity, including causing damage to testicular structure and reducing sperm count and motility. In this study, we quantified the cellular composition in the seminiferous tubules in a control group and those treated with PS microplastics of different sizes. We found that both nanoscale and microscale particles could cause decreases in spermatocytes, which could be the main cause of the sperm count reduction in our previous study [23]. The number of other cell types did not significantly change. The ratio of Leydig cells to seminiferous tubules is mainly affected by the reduction in the number of seminiferous tubules. Therefore, it seems that exposure to PS-MPs and PS-NPs predominantly harms spermatocytes. 

We analyzed the mRNA expression data of GC-2 cells in the control, PS-NP, and PS-MP groups at different doses and identified 1794 changed common genes across the different doses in the PS-NP group and 1433 altered common genes across the different doses in the PS-MP group. To better understand the DGEs of the PS-NP and PS-MP groups involved in possible functions and pathways, we analyzed the GO function and KEGG pathway enrichments of the DGEs. The results of the enrichment analyses showed that the DEGs were primarily related to biological processes such as “apoptosis”, “glycolytic process”, and “proliferation”, which is consistent with our previous findings [23]. High-quality sperm are associated with greater levels of glycolysis-derived metabolites [40]. A recent study found that PS-NPs affect glycolysis by adsorbing pyruvate kinase M (PKM) in THP-1 cells [41]. Additionally, maternal exposure to PS-MPs disrupted placental metabolism, and pathway analysis identified changes in the glycolysis/gluconeogenesis pathways [42]. It has been reported that human lung cells exposed to PS-MPs with an environmental contaminant undergo inhibition of cell proliferation [43]. However, the impact of PS-NP and PS-MP exposure on the glycolysis and proliferation of spermatogenic cells has not been reported yet. Simultaneously, the DEGs in the PS-NP and PS-MP groups were significantly related to pathways such as the PI3K-Akt signaling pathway, the TGF-beta signaling pathway, and Th1 and Th2 cell differentiation. The PI3K/AKT signaling pathway is crucial for regulating cell growth, metabolism, survival, and proliferation and plays a significant role in maintaining cellular stability and basic functions [44]. Previous studies have demonstrated that PS-MPs of different sizes induce apoptosis and necroptosis in the liver through the PTEN/PI3K/AKT/autophagy axis [45], and Di-(2-Ethylhexyl) phthalate (DEHP) and MPs induce neuronal apoptosis through the PI3K/AKT pathway [46]. MP/NP exposure can lead to dysregulation of the PI3K-AKT signaling pathway in a kidney–testes microfluidic platform [28]. In addition, PS-MPs promote the progression of inflammation and fibrosis in diabetic nephropathy through the TGF-β1/Smad2/3 signaling pathway [47]. Recent research suggests that PS-MPs may cause more harmful effects on differentiation than the proliferation of A549 and Caco-2 cells [48]. Whether PS-NPs and PS-MPs can affect spermatogenesis through these signaling pathways remains unclear. 

At present, most studies on microplastic toxicity have focused on micrometer-scale particles. Nanoplastics could pose a greater risk than microplastics due to their ability to easily penetrate biological membranes. Compared to microplastics, various nanoparticles exhibit size-dependent toxicity, with smaller sizes leading to more adverse effects in bioassays [49]. However, the toxicity of nanoplastics remains poorly studied. Thus, we further investigated the exact effects of nanoplastics on spermatocytes and the toxic differences in the molecular characteristics and pathways between nanoplastics and microplastics. The DEGs in the PS-NP group were specifically enriched in these GO entries mainly concentrated in “retinol metabolic”, “retinoic acid metabolic”, “cell cycle”, and “the process of fertilization” and enriched in the KEGG pathways mainly focused on “cell adhesion”, “Rap1 signaling pathway”, and “metabolism of cofactors and vitamins”. Retinoic acid (RA), a derivative of retinol (vitamin A), is critical to producing sperm in mammals. During fetal development, germ cells in the testes do not yet initiate meiosis, as they are insulated from RA and undergo cell cycle arrest. Recent studies suggest that the RA levels in adult testes change periodically to not only initiate meiosis but also to ensure that spermatogenesis is precisely organized for the prodigious output of sperm [50]. In our study, we found that serum retinoic acid significantly decreased in the PS-NP group but not in the PS-MP group. The retinoic-acid-metabolism-related gene Cyp26b1 was abnormally expressed at low levels in the GC-2 cells in the PS-NP group (Figure 7A,B). However, the expression of Cyp26b1 was significantly increased in the PS-NP and PS-MP groups in testis tissue (Figure S1A). Cyp26b1 is a retinoic-acid-degrading enzyme whose activity within germ cells is essential for the normal progression of spermatogenesis, and its loss can result in reduced male fertility [51]. As an active vitamin A derivative, retinoic acid metabolism efficiency determines spermatogenesis. We will further investigate the retinoic acid metabolism of the testes after exposure to PS-NP in our next study.

Since the first study in 2019 reported that exposure to polystyrene microplastics causes reproductive toxicity, numerous studies have reported on the male reproductive and developmental toxicity of microplastics on a micrometer scale in recent years [16,17,52,53,54,55,56,57,58]. However, there are still great challenges in identifying the mechanisms underlying the toxicity of polystyrene microplastics of different sizes. In our study, the number of spermatocytes significantly decreased in both the PS-NP group and the PS-MP group. The GO entries were specifically enriched and mainly concentrated in “germ cell development”, “pyruvate metabolic”, “thyroid hormone metabolic”, and “iron ion homeostasis” in the PS-MP group. During the entire spermatogenesis process, the Sertoli cells produce a substantial amount of pyruvate, which they then pass to spermatogenic cells at all levels. Pyruvate is particularly important for the ATP required for meiosis in primary spermatocytes and sperm cells [59]. Pyruvate can be transformed into lactate in vivo by lactate dehydrogenase. Lactate dehydrogenase B (LDH-B) transcripts can be found in spermatogonia and primary spermatocytes, and the LDH-B gene is expressed mainly in germ cells that are situated close to the basement membrane, which is essential for normal spermatogenesis [60]. Our study demonstrated that LDH-B was significantly downregulated after PS-NP and PS-MP exposure. The encoding gene Dhtkd which 1,2-Oxoadipate dehydrogenase (OADH), was only significantly downregulated in the PS-MP group. These data indicate that alterations in the expression of specific cellular genes may alter the pyruvate metabolism of spermatocytes at different levels in nano- and micro-sized microplastics. Alterations of pyruvate metabolism mainly contribute to spermatogenesis impairment after PS-MP exposure. Thyroid hormones also play an important role in spermatogenesis. Proper thyroid levels in the testes are essential for normal testicular spermatogenesis and maintenance of reproductive function. Thyroid abnormalities (hyperthyroidism or hypothyroidism) can affect testicle size and the proliferation and differentiation of germ cells, thereby affecting spermatogenesis and fertility [61]. In our study, the thyroid-hormone-metabolism-related genes were downregulated after PS-NP and PS-MP exposure, especially thyroid hormone receptors encoding the Thra gene (Figure 7H). However, the expression of Thra only significantly decreased in the PS-MP group (Figure S1E). It is suggested that PS-MP exposure may cause alterations in thyroid hormones due to defective hormone receptors. Ferroptosis is a type of programmed cell death mediated by iron-dependent lipid peroxidation, leading to excessive lipid peroxidation in different cells [62]. Iron is essential for spermatogenesis and male reproductive function. The latest study revealed that the synergistic effect of PS-MPs and Cd on male reproductive toxicity, which included decreased sperm parameters, impaired spermatogenesis and damaged testicular structures, which is attributed to testicular ferroptosis [63]. Nevertheless, the role of ferroptosis in the toxicity of different sizes of MPs is still obscure. In our study, the ferroptosis pathway was only enriched in the PS-MP group, suggesting micro-sized MPs may cause an extreme iron ion homeostasis disorder. 

## 5. Conclusions

This study revealed the effect of PS-NPs and PS-MPs on spermatogenesis in mice and analyzed common and special molecular characteristics and pathways for the first time. Exposure to PS-NPs and PS-MPs decreased the number of spermatocytes in the seminiferous tubules to impair spermatogenesis. Independent validation revealed that the expression of the selected key specific DEGs was highly consistent with RNA-seq data, suggesting the same effect was caused by microplastics of different sizes through different molecular mechanisms. Alteration of the retinoic acid metabolism pathway was the reason for the decrease in spermatocytes in the PS-NP group, which was also confirmed by the significant decrease in the Cyp26a1 expression level. Simultaneously, the alternation of thyroid hormone metabolism caused a decrease in the number of spermatocytes in the PS-MP group, which was also confirmed by the significant decrease in the Thra expression level. The limitations of the present study may include the following aspects. First, we did not detect the accumulation of PS-NPs and PS-MPs after treatment due to limitations in detection technology. Second, the selected key specific DEGs and potential molecular mechanisms require further exploration. Third, we did not detect the specific expression in the testes to further identify the number of changes in each cell type. Nonetheless, our findings provide a warning regarding the reproductive toxicity of microplastics of different sizes and offer novel insights into the toxicity mechanisms of PS-NPs and PS-MPs. 

## Figures and Tables

**Table 1:** Primer sequences of the target genes.

Gene	Sequences (5′–3′)	
	Foreword	Reverse
*Dhtkd1*	5′-CCTCGCTCTCCTTGGTTGTAG-3′	5′-CCTCAGTTGACCATGGCCTT-3′
*Ldhb*	5′-GGATTCACCCCGTGTCTACC-3′	5′-GAAGGACGATGAGGTCGCTC-3′
*Slc16a2*	5′-CCCATTGGTTCTTGGCTCTGA-3′	5′-GGGTAGGGGGCAGAATAAGT-3′
*Thra*	5′-TTGCGTGCTGTTTCCCCATA-3′	5′-AACAAATCGAGGGGCCAGAG-3′
*Cyp26a1*	5′-TCTGGGACCTGTACTGTGTGA-3′	5′-AAGCCGTATTTCCTGCGCTT-3′
*Cyp26b1*	5′-TGCCCATACCCCATCGCC-3′	5′-GGCTGCGAGGTGATCGAAGA-3′
*β-actin*	5′-GTCCCTCACCCTCCCAAAAG-3′	5′-GCTGCCTCAACACCTCAACCC-3′

**Table 2:** The number of common and specific DEGs in the different groups.

Size	Common	10 μg/mL	20 μg/mL	40 μg/mL
NPs	1794	2176	1338	1762
MPs	1433	1766	1410	2073

## Data Availability

Data are available from the authors upon request.

## Supplementary Materials

The following supporting information can be downloaded at https://www.mdpi.com/article/10.3390/biomedicines12081714/s1. Tables S1–S4: The original date of DEG screening analysis and functional analysis of DEGs. Figure S1: The relative expression of characteristically genes in the testes tissue.
