# Supplementary material for: The Potential Mechanisms Involved in the Disruption of Spermatogenesis in Mice by Nanoplastics and Microplastics
Source: Biomedicines. 2024 Aug 1;12(8):1714. doi: 10.3390/biomedicines12081714 (PMC11351746; doi:10.3390/biomedicines12081714)
Supplement: Supplementary file 1 [file biomedicines-12-01714-s001.zip › Figure S1.pdf]

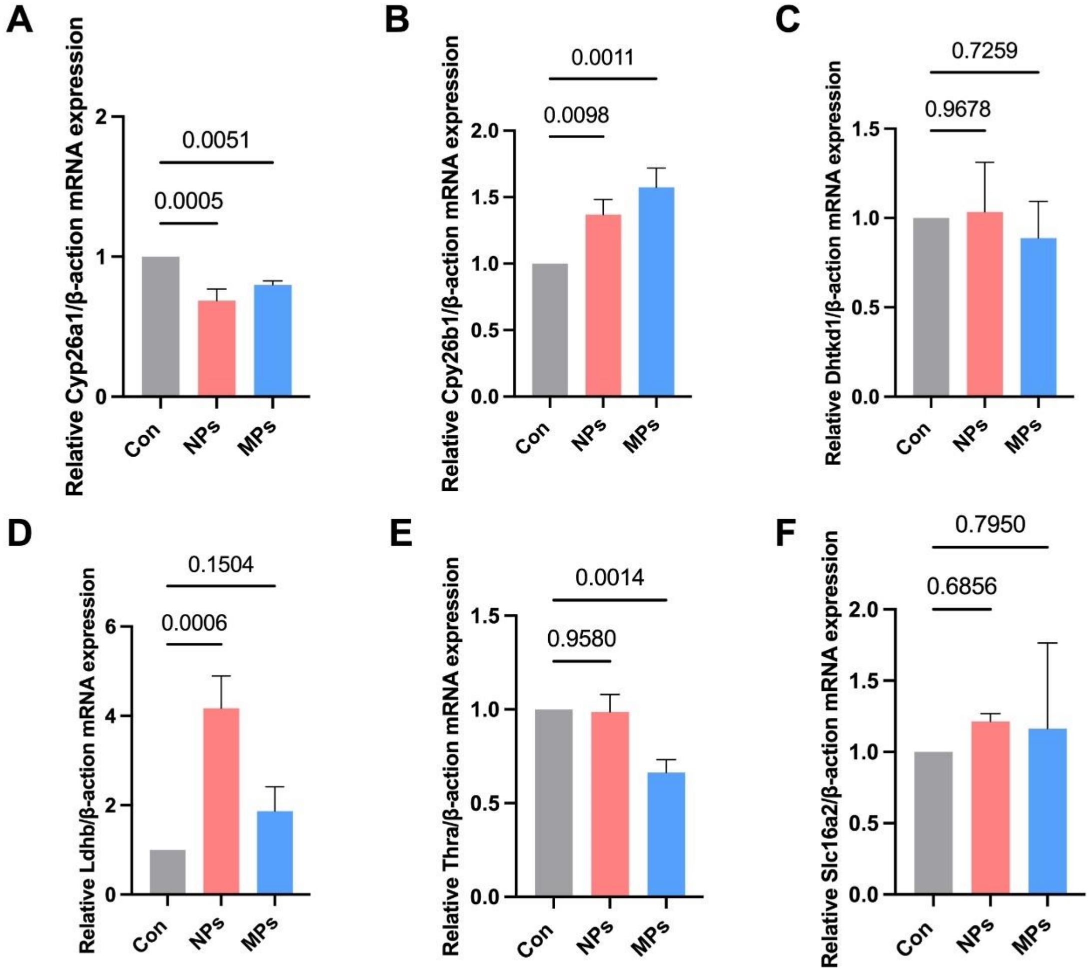

Figure S1. (A) Relative expression of Cyp26a1 mRNA in testis tissue exposure to PS-NPs and PS-MPs. (B) Relative expression of Cyp26b1 mRNA in testis tissue exposure to PS-NPs and PS-MPs. (C) Relative expression of Dhtkd1 mRNA in testis tissue exposure to PS-NPs and PS-MPs. (D) Relative expression of Ldhb mRNA in testis tissue exposure to PS-NPs and PS-MPs. (E) Relative expression of Thra mRNA in testis tissue exposure to PS-NPs and PS-MPs. (F) Relative expression of Slc16a2 mRNA in testis tissue exposure to PS-NPs and PS-MPs.
